# Predators can facilitate herbivory in nutrient-limited marine ecosystems

**DOI:** 10.1038/s41598-025-34145-6

**Published:** 2025-12-30

**Authors:** Anish Paul, Harshul Thareja, Rohan Arthur, Teresa Alcoverro, Sandeep Pulla, Rucha Karkarey

**Affiliations:** 1 Wildlife Biology and Conservation Program, National Centre for Biological Sciences - Tata Institute of Fundamental Research, Bengaluru, India; 2 Ecology and Evolution Group, National Centre for Biological Sciences - Tata Institute of Fundamental Research, Bengaluru, Karnataka India; 3 Lancaster Environment Centre, Lancaster University, Lancaster, UK; 4 Oceans and Coasts Programme, Nature Conservation Foundation, Mysore, India; 5 Centre D’Estudis Avançats de Blanes (CEAB – CSIC), Blanes, Spain

**Keywords:** Ecosystem functions, Nutrient stoichiometry, Bottom-up processes, Predator-prey interactions, Mesopredator release, Coral reefs, Ecology, Ecology, Ocean sciences

## Abstract

Predators influence ecosystem functioning through consumptive and non-consumptive effects. Recent studies suggest that predators can also be an essential source of limiting nutrients in ecosystems such as coral reefs, potentially influencing prey ecology through nutrient input via their excreta. With rising commercial fishery, mesopredatory fishes are being selectively harvested from reefs. Yet, there is incomplete knowledge of the consequences of this extraction on essential ecosystem processes. Using field experiments and observations, we examined how mesopredatory fishes influence herbivory along a fishing-induced mesopredatory fish biomass gradient in the Lakshadweep Archipelago in the northern Indian Ocean. We found that mesopredatory fish excreta have greater proportion of phosphorus than nitrogen. Along the gradient, primary and secondary productivity increased, after accounting for pelagic nutrient subsidies. Further, herbivory rates increased with increasing mesopredator biomass, while prey anti-predator response remained unchanged. Our results suggest that mesopredator-induced alterations of nutrient stoichiometry stimulate primary and secondary productivity and enhance herbivory in phosphorus-limited coral reefs, particularly in systems experiencing mesopredator release following selective fishing of apex predators. Our study shifts focus from the traditional top-down role of predators, highlighting an overlooked bottom-up pathway by which mesopredators can influence ecosystem functioning. Global decline of predators could modify ecosystem processes in ways that are yet unknown, leaving them increasingly vulnerable to future disturbances.

**Supplementary Information:**

The online version contains supplementary material available at 10.1038/s41598-025-34145-6.

## Introduction

 Predator-prey interactions are a major governing process in natural ecosystems^1,2^. Predators can directly influence the size, demography, and population structure of prey, as well as influence the community composition of an ecosystem^1–4^. Apart from directly influencing the prey population through predation, predators also generate a ‘landscape of fear’^5^, where prey species must remain vigilant of an imminent attack not only in the presence of predators but even in their absence^6,7^. However, the consumptive and non-consumptive top-down effects of predators and mesopredators are not prominent across all ecosystems^8,9^, and predator-mediated trophic cascades are considered to be ‘exceptions rather than rule’ in ecosystems with complex trophic structures^10^.

Besides exerting top-down effects, predators can also influence bottom-up processes by mediating cross-ecosystem nutrient transfers or by altering the stoichiometry of nutrients available to primary producers^11–14^. These effects may be particularly evident in oligotrophic and mesotrophic ecosystems where nutrient availability limits productivity. In such systems, consumer-derived nutrients could modulate primary productivity and, subsequently, consumption, i.e., herbivory^11,12,15–18^. However, much of our current understanding of top predators’ influence on ecosystem processes comes from terrestrial and pelagic ecosystems, where their lower abundance and biomass relative to lower trophic levels may limit their nutrient-mediated effects^6,7,19–25^. In contrast, in ecosystems where predators, particularly mesopredators, constitute a high proportion of total biomass, they can potentially influence the rates of ecosystem processes through nutrient-mediated pathways^26–29^. This potential facilitation of productivity through predatory fish-derived limiting nutrients has rarely been explored, with only a few examples from seagrass ecosystems and low-diversity coral reefs in the Caribbean^11,28^. Additionally, while the role of predators in structuring herbivory through top-down effects on herbivore populations has been well documented across ecosystems^23,30–33^, how predator-derived nutrients could influence herbivory, through their effects on primary productivity, remains underexplored^34,35^.

Mesopredatory fishes constitute a significant proportion of the standing biomass in undisturbed reefs, often much greater than any terrestrial system, and frequently form permanent or temporary aggregations to feed and/or reproduce^26–29,36,37^. However, the complexities of food webs in coral reefs, associated with uncertainties in their trophic position and diet inconsistencies, have made the role of predators and mesopredators in generating trophic cascades in coral reefs ambiguous and highly context-specific^10,38–43^. While predator removal has been reported to promote benthic recovery^43^, studies have also reported the co-occurrence of high coral cover and high predator biomass in reefs^44,45^. Additionally, owing to their diet, excretory inputs from mesopredatory fish are a major source of phosphorus — one of the limiting nutrients in many coral reefs of the world — that can determine algal productivity^11,18,46,47^. Thus, in the absence of strong consumptive and non-consumptive effects of predation, mesopredators can potentially mediate herbivory levels in coral reefs by increasing algal production, thereby increasing the algal-removal potential of herbivores through nutrient-mediated pathways^11,13,26,48^. In coral reefs, herbivory is a key process in maintaining the stability and health of the ecosystem^49–51^, as it confers resilience to reefs by removing algae that compete with coral recruits for substrate, nutrients, growth, reproduction, and survivorship^51–54^.

Unpacking these nuanced trophic relationships becomes particularly important in the light of increasing fishing impacts on reefs. As true apex predators, such as sharks, decline due to overfishing, mesopredatory species, like groupers, snappers, and emperors, are attaining the role of top predator and are also increasingly becoming fisheries targets. We lack the understanding of how their removal will impact essential ecosystem processes in coral reefs^55,56^. It is therefore crucial to investigate the influence of mesopredatory fishes on algal production and herbivory levels in reefs, especially where they can play essential bottom-up roles as nutrient providers in nutrient-limited systems.

The islands of the Lakshadweep Archipelago are ideally suited to test the role of mesopredators in influencing bottom-up and top-down processes in nutrient-limited ecosystems. Some reefs of Lakshadweep have been relatively unfished until recently^57,58^. Nutrient-limited waters and the relatively lightly fished fish community of some of the Lakshadweep’s coral atolls make it a suitable system to study the effect that mesopredatory fishes may have on herbivory levels by altering the bottom-up pathways through nutrient input. We employed a combination of experimental and observational methods to quantify herbivore and piscivore biomass, the stoichiometry of nutrient contributions by herbivores and mesopredators (based on biomass and species-specific nutrient input rates), algal growth rates, herbivore productivity, herbivory rates and prey anti-predatory behaviour along a fishing-induced mesopredatory reef fish biomass gradient to evaluate support for bottom-up vs. top-down processes in the coral reefs of Lakshadweep. The specific questions we addressed were:

- Do primary and secondary productivity increase with increasing mesopredator biomass in a nutrient-limited coral reef ecosystem?
- Do herbivory rates vary in response to variations in mesopredator biomass?
- Is there evidence for top-down non-consumptive effects in the form of increasing vigilance with increasing mesopredator biomass?

## Results

### Do primary and secondary productivity increase with increasing mesopredator biomass in a nutrient-limited coral reef ecosystem?

#### Consumer-derived nutrients

**Fig. 1:**
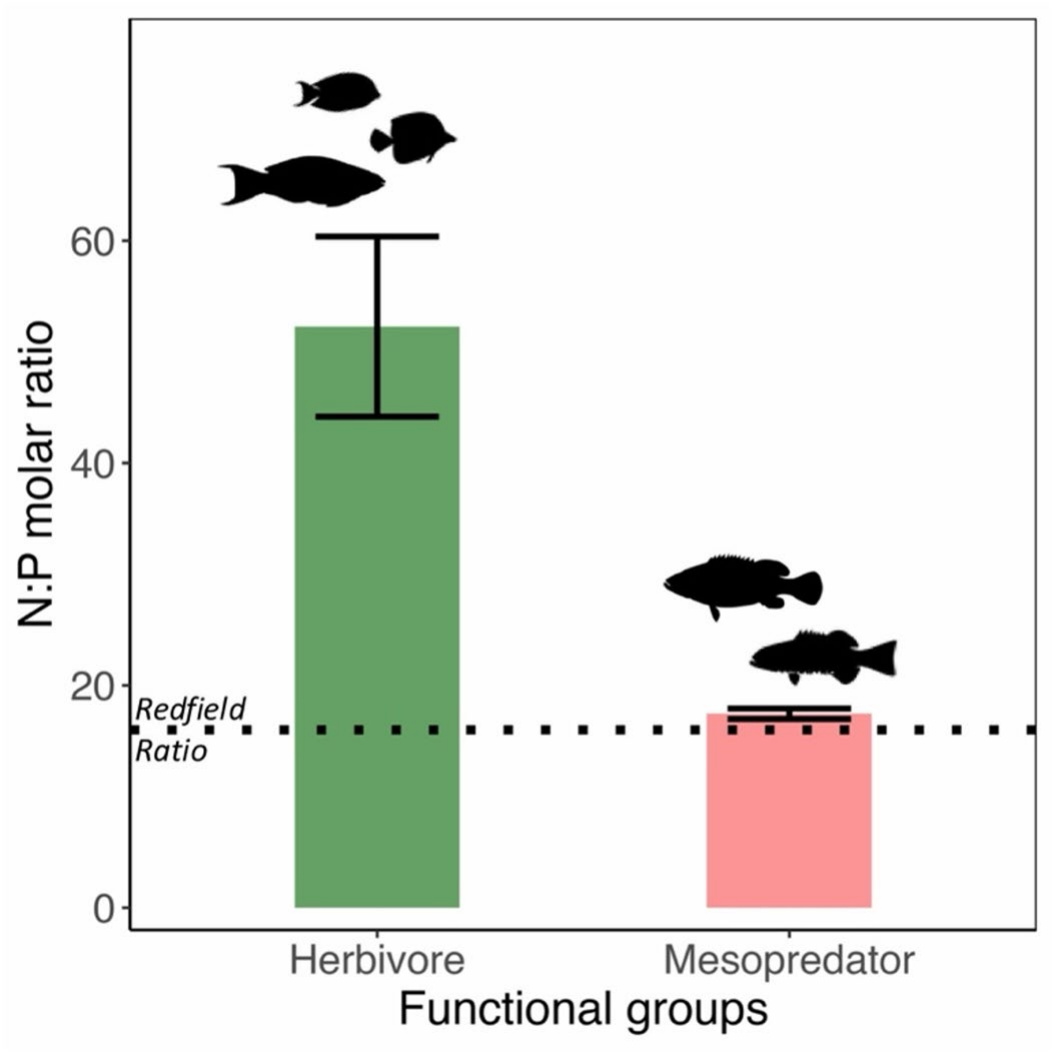
Nitrogen to Phosphorus molar ratio comparison between herbivore and mesopredator excreta across sites. Error bars represent bootstrapped standard error around the mean. The dotted line represents the classical Redfield Ratio (16:1).

The mean molar ratio of nitrogen to phosphorus across sites was 52.3:1 (± 8.1, SE) for herbivore excreta, whereas it was considerably lower, at 17.5:1 (± 0.47, SE) for mesopredator excreta (Fig. 1).

#### Primary productivity

**Fig. 2:**
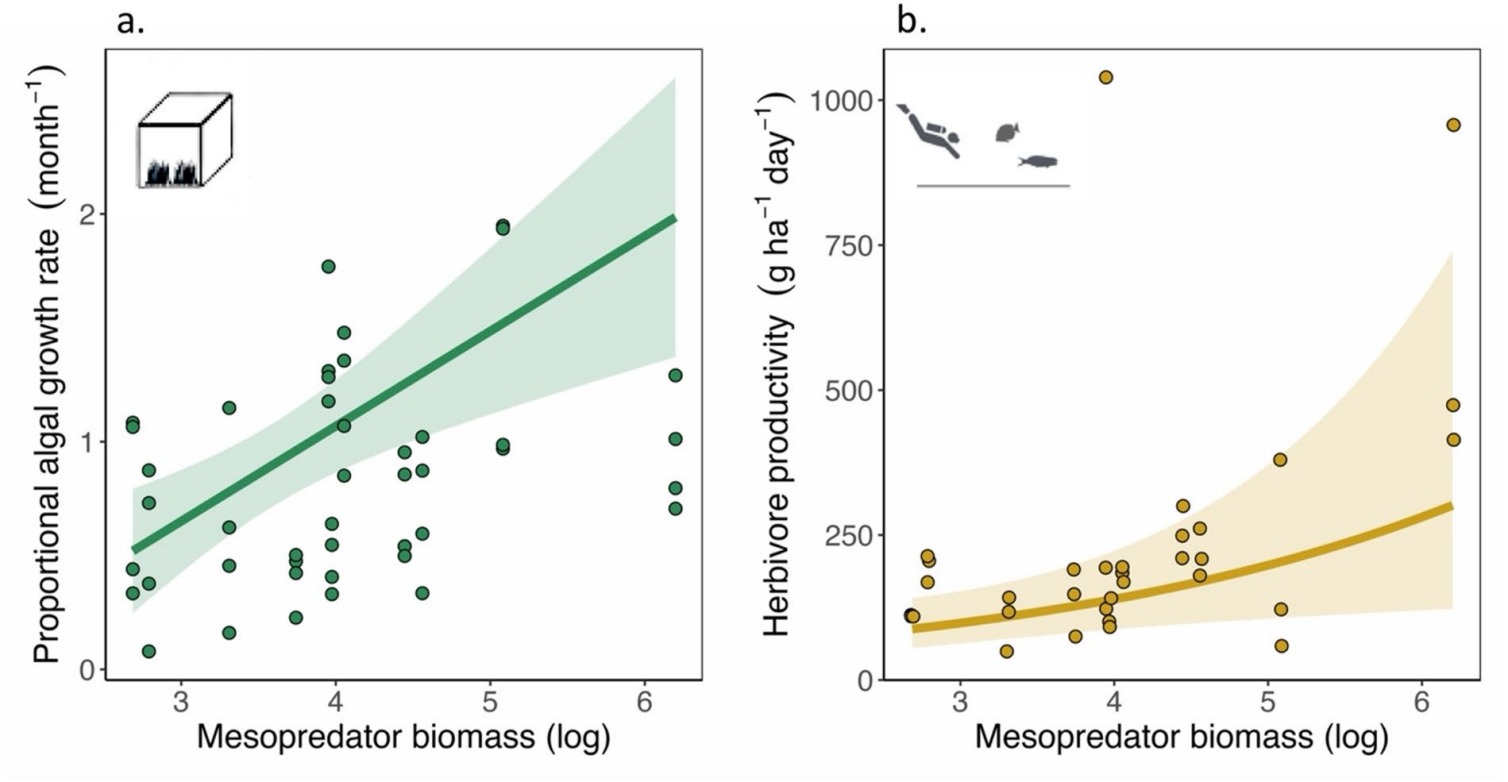
Primary and secondary productivity increases with mesopredator biomass. (**a**) Algal growth rate was measured as the monthly average increase in algal frond heights in the absence of herbivory. Algal growth rate increases with increasing log-transformed mesopredator biomass. Points represent observed data (*n* = 44), and lines represent the partial predicted values from the linear mixed-effects model, with all other predictors set at their mean. The shaded regions represent the 95% confidence intervals. (**b**) Herbivorous fish productivity was estimated using species identity, size and abundance of grazers, scrapers and excavators obtained from the underwater visual census. Herbivorous fish productivity shows a positive correlation with log-transformed mesopredator biomass. Points represent observed data (*n* = 33), and lines represent the partial predicted values from the generalized linear mixed-effects model with gamma error distribution and log-link, with all other predictors set at their mean. The shaded regions represent the 95% confidence intervals.

Primary productivity was measured as the monthly proportional growth rate (change in turf height per month/initial height of turf) of turf algae inside herbivore exclosures. Proportional algal growth rate increased by 0.41 month^− 1^ (95% CI 0.18 to 0.63, *p* = 0.001) per unit standard deviation increase in log-transformed values of mesopredator biomass (Fig. 2a, Supplementary Material 1). In contrast, proportional algal growth rate was not significantly influenced by herbivore biomass (Supplementary Material 1). Physical aspect of the site, i.e., the position of the site with respect to the north-south orientation of the atolls, also significantly influenced algal growth rate, with the western aspect – which is more exposed to the south-west monsoon – showing a reduction in proportional growth rate by by 0.61 month^− 1^ (95% CI 0.24 to 0.99, *P* = 0.002) (Supplementary Material 1, 2).

#### Secondary productivity

Herbivorous fish productivity, i.e., secondary productivity, increased by a factor of 1.41 (95% CI 1.07 to 1.85, *p* = 0.013) per unit standard deviation increase in log-transformed values of mesopredator biomass (Fig. 2b, Supplementary Material 3). Secondary productivity also increased by a factor of 1.40 (95% CI 1.08 to 1.81, *p* = 0.010) with an unit standard deviation increase in structural complexity of the site (Supplementary Material 3). Resource availability and aspect of the site had no statistically significant relationship with herbivore productivity (Supplementary Material 3).

**Fig. 3:**
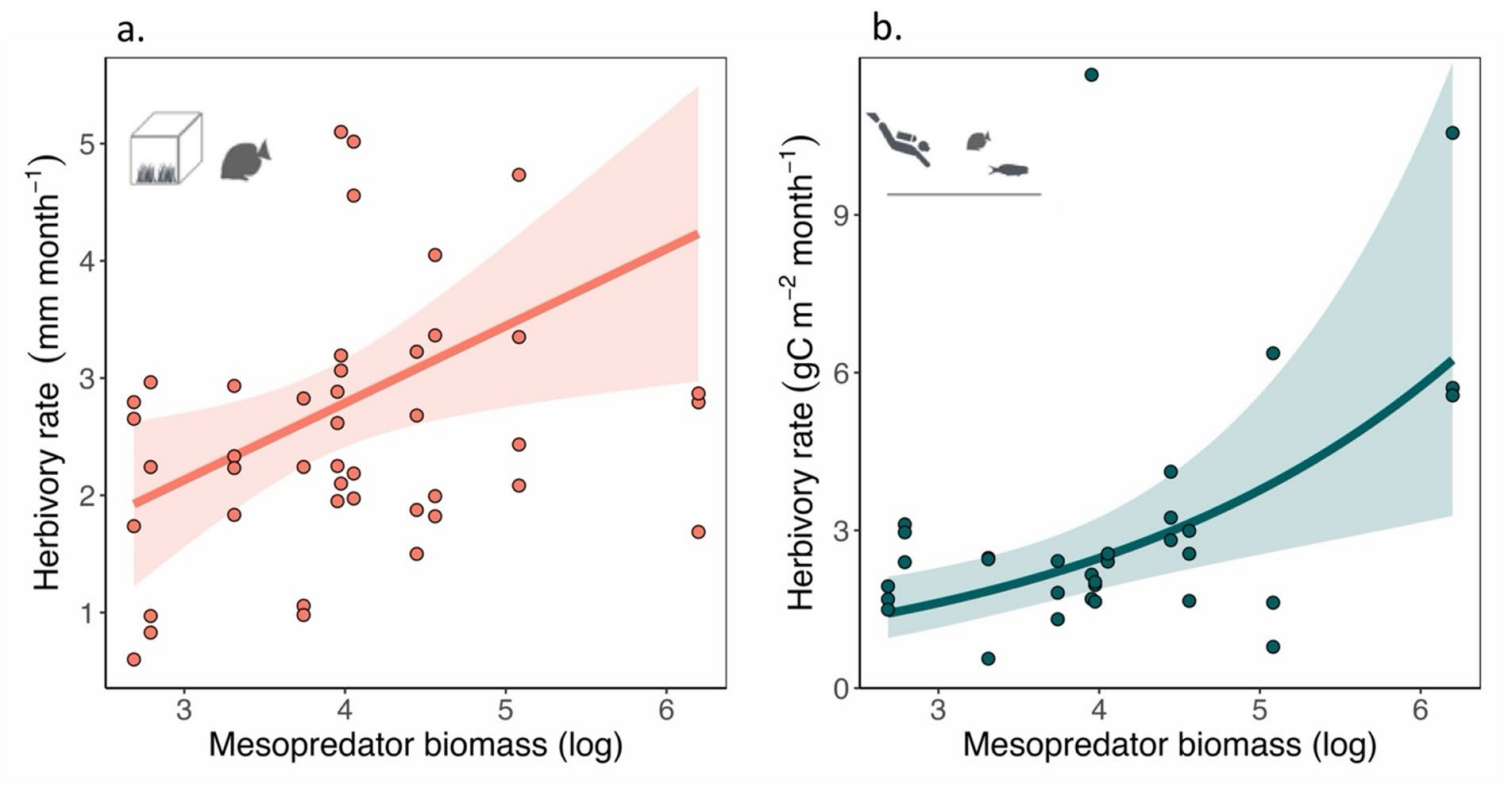
Herbivory rates increase along the mesopredator biomass gradient. We employed two complementary approaches to estimate herbivory: (**a**) Site-level Herbivory rates were measured as the difference between monthly algal growth inside the exclosures and that of the paired open herbivory plot located next to it. Site-level herbivory rate positively correlates with log-transformed mesopredator biomass. Points represent observed data (*n* = 44), and the line represents the partial predicted values from the linear mixed-effects model, with all other predictors set at their mean. The shaded region represents the 95% confidence interval. (**b**) Community-wide herbivory rates, measured in terms of grams of carbon ingested per unit area by the herbivorous fish community, shows a positive correlation with log-transformed mesopredator biomass. Points represent observed data (*n* = 33), and the line represents the partial predicted values from the generalized linear mixed-effects model with gamma error distribution and log-link, with all other predictors set at their mean. The shaded region represents the 95% confidence interval.

### Do herbivory rates vary in response to variations in mesopredator biomass?

#### Reef-wide herbivory rate

Reef-wide herbivory rate was calculated as millimeters of algal frond lost per month from 20 cm × 20 cm plots. Mesopredator biomass had a positive influence on herbivory rate, with a unit standard deviation increase in the log of mesopredator biomass associated with a 0.64 mm/month increase in algal frond loss (95% CI 0.12 to 1.16, *P* = 0.017) within a 20 cm × 20 cm plot (Fig. 3a, Supplementary Material 4). Herbivore biomass, percentage algal cover, and aspect had no statistically significant influence on herbivory rates (Supplementary Material 4).

#### Community-level herbivory rate

The community-level herbivory rate, calculated using the underwater visual census data, increased by a factor of 1.51 (CI 1.18 to 1.93, *p* = 0.001) per unit standard deviation increase in log-transformed mesopredator biomass (Fig. 3b, Supplementary Material 5). Herbivory rates also increased by a factor of 1.30 (CI 1.03 to 1.65, *p =* 0.028) per unit standard deviation increase in structural complexity of the site (Supplementary Material 5). Percentage algal cover and aspect of the site had no statistically significant relationship with community-level herbivory rates (Supplementary Material 5).

**Fig. 4:**
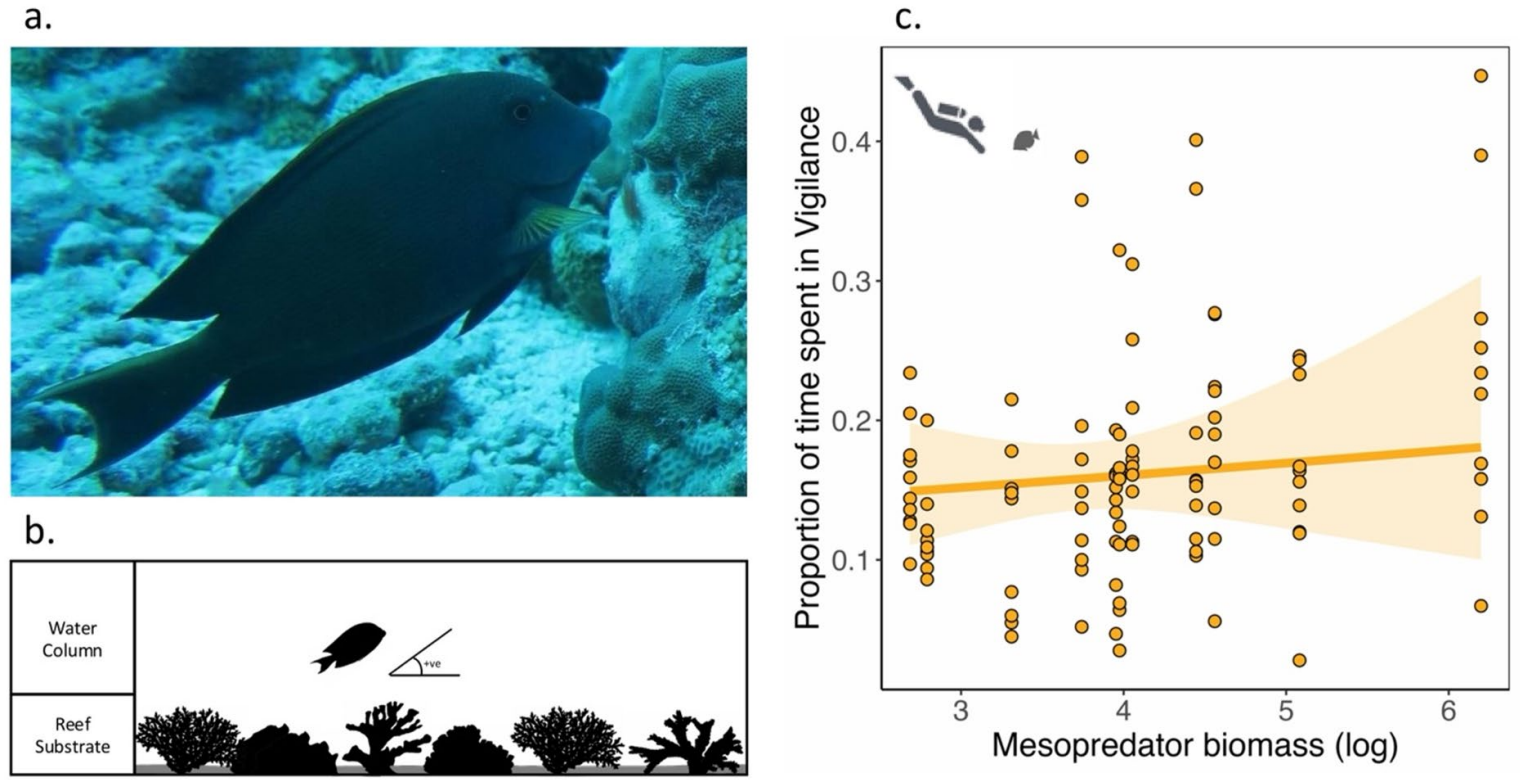
Time spent in vigilance by individuals of a hyperabundant prey species remains unchanged along the mesopredator biomass gradient. (**a**) We conducted focal animal observation (*n* = 110) on a candidate prey species, *Ctenochaetus striatus*, to understand extent of the presence of non-consumptive effects of mesopredators on prey individuals. (**b**) Vigilance was defined as the behavioural state when the face of the focal individual is directed toward the water column at an upward angle away from the reef substrate. (**c**) Proportion of time spent in vigilance by *C. striatus* individuals shows no statistically significant relationship with increasing log-transformed mesopredator biomass. Points are observed data and lines represent the partial predicted values from the generalized linear mixed-effects model with all other predictors set at their mean. The shaded regions represent the 95% confidence intervals.

### Is there evidence for top-down non-consumptive effects in the form increasing vigilance with increasing mesopredator biomass?

We conducted 3-minute focal animal observations on individuals of a hyperabundant prey species, *Ctenochaetus striatus* (Fig. 4a), to quantify proportion of time spent in exhibiting anti-predatory behaviour, i.e., vigilance, by individuals along the mesopredator biomass gradient (Fig. 4b). None of the predictors, including mesopredator biomass, had any statistically significant relationship with the proportion of time spent in vigilance by *C. striatus* individuals (Fig. 4c, Supplementary Material 6).

## Discussion

Mesopredators input key nutrients into reefs^13,17,28,59^, which can enhance algal productivity^34^. Our results indicate that mesopredators can also indirectly enhance reef-scale herbivory via nutrient-mediated pathways and may play a key role in the functioning of coral reef ecosystems.

Coral reefs can vary in their degree of nutrient-limitation based on their origin and substrate characteristics^46,47,60,61^. Waters around coral atolls with calcium carbonate substrate tend to generally be nutrient poor and studies indicate that coastal ecosystems of the Chagos-Laccadive ridge are likely to be phosphorus-limited^46,47,62–64^. Furthermore, experimental evidence from the Bahamian reefs, which also have a calcium carbonate skeleton, suggests that microalgal productivity is phosphorus-limited in such systems^11^. Our results suggest that mesopredatory fishes supply nutrients in reefs at an N: P molar ratio closer to the classical Redfield Ratio of 16:1 and much lower than that of herbivorous fishes^65^. This suggests that there is greater proportional phosphorus than nitrogen in mesopredator excreta, which can thus influence primary productivity in a phosphorus-limited system^65^. This conforms with studies from other parts of the world, establishing mesopredator excreta as a critical source of phosphorus in nutrient-limited tropical marine systems^11,66^.

Consistent with the previous studies from Lakshadweep, physical aspect of the site had the greatest effect on algal growth rates, with sites located on the western aspect having a relatively lower growth rate than the ones located on the east^67^. In our study, aspect stands as a proxy of wave exposure regimes in Lakshadweep, which likely mediates the availability of pelagic nutrient subsidies — another essential source of nutrient for reef productivity — transported to the reef by subsurface currents^68,69^. However, our results indicate a significant positive relationship between primary productivity and mesopredator biomass after statistically controlling for pelagic nutrient subsidies through aspect. Our results thus suggest that, aside from their traditional role in mediating top-down processes, mesopredatory fishes can potentially enhance algal growth rate, i.e., primary productivity, in nutrient-limited atoll systems. This highlights the role of predator-derived nutrients and thus, predators, in influencing bottom-up pathways^11^.

Consistent with our expectations, herbivore productivity increased with mesopredator biomass in the reefs of Lakshadweep^34,35^. In our estimation of herbivore productivity, we only included herbivorous reef fishes with body sizes greater than 10 cm. This body size far exceeds the average size of prey (1.75 cm, 95% CI 0.8 to 3.65) in coral reefs^70^. Therefore, the threat of predation on the sampled herbivorous fish is likely to be insignificant. Hence, it indicates that the observed herbivore productivity is unlikely to influence the observed mesopredator biomass in our study, as this productivity is not accessible to the mesopredators for consumption. Additionally, the targeted fishing pressure towards mesopredatory reef fishes in the atolls of Lakshadweep leaves the herbivorous fish community relatively undisturbed^71^, which in turn reduces the possibility of fishing pressure confounding the relationship between herbivore productivity and mesopredator biomass. Thus, we suggest that the positive correlation between mesopredatory fish biomass and herbivorous fish productivity can potentially be caused by indirect facilitation of herbivory by piscivorous fishes, where the nutrient-enriched seascape generated by predator-derived nutrients (primarily increased phosphorus availability in a phosphorus-limited ecosystem) leads to greater primary productivity, and thus enhances secondary productivity^35,72^. Similar patterns have been observed in the reefs of Australia, where novel fish communities, resulting from tropicalization, increased the availability of turf algae, which in turn increased herbivore productivity^72^. Studies from similar oceanic atolls in the Seychelles support a similar hypothesis, where enhanced nutrient availability from seabird excreta has increased secondary productivity via increased turf growth rates (primary productivity)^34^. While nutrient input from reef fishes is expected to be much less compared to that of seabirds^14,15,26^, studies on grunts (Haemulidae) and damselfishes (Pomacentridae) have shown that fish-derived nutrients can increase algal and coral growth rates on a smaller spatial scale^28,73,74^. However, further studies examining isotopic signatures in algae and fish tissues, fine-scale oceanographic patterns, and experimentally manipulating consumer biomass, nutrient availability and stoichiometry are required to definitively validate the potential indirect effects of mesopredators on herbivore productivity in coral reefs.

Herbivory is considered to be a critical ecosystem function in the coral reefs^75,76^. Spatial patterns of herbivory are known to have dramatic influences on the structure, composition and distribution of plant and algal communities in both terrestrial and marine environments^77–82^. Our results indicate a statistically significant positive correlation between mesopredator biomass and herbivory rates.

Although mesopredators strongly dictate herbivorous fish demography through direct predation during their juvenile state^83,84^, predation threat on herbivores reduces significantly with increasing body size^70,85,86^. This suggests a negligible direct consumptive effect of piscivores on observed herbivores (body size ≥ 10 cm) in our study. However, the non-consumptive effects of predators can alter feeding behaviour of prey even when the prey species, owing to their body size, do not experience any imminent predation threat^7,31,87,88^. Increased perceived predation threat can increase rates of feeding by individuals^31^, thus potentially leading to greater consumption, i.e., herbivory at the level of the reef. However, presence of non-consumptive effects on fishes of lower trophic groups is known to be weak^8,9^. Our focal fish observations of a hyperabundant primary consumer species in Lakshadweep, *Ctenochaetus striatus*, further evidenced this. Along the mesopredatory fish biomass gradient, the adult *C. striatus* individuals exhibited no change in anti-predatory behaviour, suggesting the absence of a strong non-consumptive effect of predation on adult individuals of prey fish guild^8,9^. Thus, we argue that observed patterns in herbivory rates along the mesopredator gradient are less likely to be driven by faster consumption of algae by herbivores as a response to perceived predation threat^31,32^. Studies suggest that herbivore biomass in coral reefs strongly correlates with turf algal productivity rather than the total available biomass of turf algae^34,72,89,90^. Hence, we argue that the observed pattern is likely due to enhanced primary productivity resulting from mesopredator-derived nutrients, which increases resource availability and consumption in reefs with greater mesopredator biomass^91^. Thus, our results indicate that in the absence of strong consumptive and non-consumptive effects on herbivores, mesopredators can potentially facilitate herbivory in nutrient-limited systems.

Establishing a definitive causal relationships between herbivory effects of predator-derived nutrients on community-wide herbivory and disentangling the effects of other factors, such as resource availability, nutrient composition of the benthos, structural complexity, sedimentation rates, local hydrodynamics and spatial configuration of sites requires further investigation using transplant experiments of algal mats and bulk or compound-specific stable isotope tracers across trophic levels^34,92–94^. However, our study draws attention to a vastly understated role of mesopredators in a system where their top-down role is often debated. It highlights the nuances of trophic interactions and ecosystem functions, aiming to improve the understanding of trophic pathways in complex ecosystems such as coral reefs. Recently, an extensive body of work has established another top trophic group of the near-shore ecosystems, the seabirds, as an integral part of nutrient dynamics in coral reefs^15–17,34,35,93^. While mesopredatory fishes can have much less quantity of nutrient input and spatial coverage than nesting seabirds and do not bring in new nutrients from external sources, we argue that they can also play an important role in coral reef nutrient dynamics due to greater proportion of phosphorus in their excreta, especially in atolls where other sources of nutrients are often limiting. Although more work is required to understand the nuances of nutrient fluxes and patterns of productivity and herbivory under varying physicochemical and ecological conditions, our results indicate that predatory fishes, specifically mesopredators, that may not exert a strong top-down influence on the adult life stages of lower trophic levels, may potentially impact the functions performed by these groups by affecting nutrient dynamics and productivity in nutrient-limited systems.

### Caveats and future research

Herbivory in coral reefs is a complex ecological process shaped by various biotic and abiotic factors and often varies across broad and fine spatial and temporal scales^67,91,95,96^. Using multiple lines of evidence, our study indicates the role of mesopredators in altering ecosystem function from bottom-up pathways^97^. While mesopredators and herbivores have very distinct nutrient stoichiometry in their excreta, species-specific differences in nutrient inputs could also play a major role in understanding functional importance of fish species in reef nutrient dynamics^98^, which can inform fisheries management of these systems. Furthermore, while we have quantified primary productivity and reef-scale herbivory using standardized methods^34,67^; the spatial coverage of our herbivory assay remains low. As herbivores can alter spatial patterns of consumption in response to perceived predation threat, future studies should examine fine-scale herbivory patterns within a site to understand how reef-characteristics mediates patterns of herbivory under differing predation threat^42,44^. Additionally, while our findings from the focal behavioural observations of *C. striatus* conform with earlier studies^8,9^ and likely represent behavioural responses of the prey guild, it is imperative to examine species-specific anti-predatory responses of true herbivores in the reefs and their contribution to herbivory. While overall herbivory levels, quantified as total algal removal from the substrate over a period of time will likely remain unchanged, the pattern and intensity of herbivory rates are likely to vary with the time of day^96,99,100^. Thus, future research should engage with understanding fine-scale temporal patterns of herbivory and anti-predatory responses of prey to holistically understand spatial and temporal patterns of herbivory under differing predation pressures. While our work lacks these finer-scale measurements and largely infers causality from correlational data, it highlights the role of predators in maintaining reef functions beyond predation itself, and offers avenues for further enquiry.

## Conclusion

Pervasive targeted fishing activities have led to a trophic downgrading in coral reefs; with apex predators like sharks being replaced by mesopredators like groupers, snappers, and emperors, which may lack similar strength of non-consumptive effects on lower trophic groups^8,9,101–103^. However, our study highlights a possible role of mesopredatory reef fishes in regulating primary productivity through nutrient input and stoichiometric alteration, and thereby, the overall herbivory rates at the level of the reef. Despite the prevailing narrative of predators influencing ecosystem processes by exerting top-down controls^31,32,87,95,100,104^, our result highlights an equally significant bottom-up influence, where mesopredators can supply the limiting nutrients and maintain levels of primary and secondary productivity in nutrient-limited systems such as coral reefs^11,13^. It also emphasizes the importance of conservation efforts targeting mesopredatory fish populations and underscores the need for sustainable fisheries management practices. Unsustainable targeted extraction of commercially important mesopredatory reef fishes can disrupt nutrient cycling and compromise primary productivity in coral reefs. This, in turn, could trigger cascading effects across trophic levels, but rather than following the traditionally known top-down pathway, these effects might originate from bottom-up processes.

## Methods

### Study site and study design

**Fig. 5:**
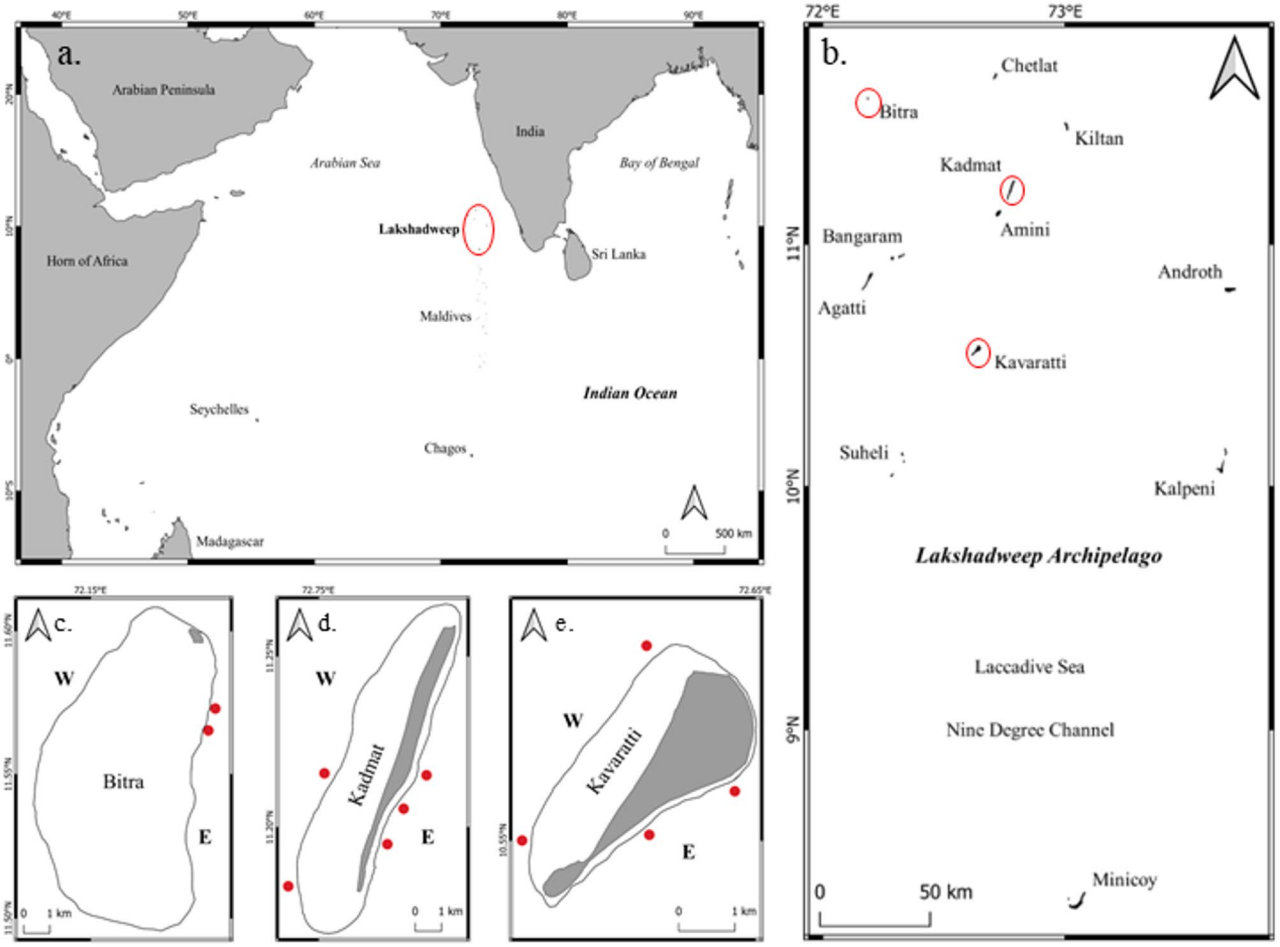
We conducted the study along a mesopredatory reef fish biomass gradient in the Lakshadweep Archipelago. (**a**) Lakshadweep Archipelago is situated in the northern Indian Ocean and is part of the Chagos-Laccadive Oceanic Ridge. (**b**) Lakshadweep comprises 12 coral atolls. We sampled 11 sites (red dots) across three atolls: (**c**) Bitra, (**d**) Kadmat and (**e**) Kavaratti, in two exposure regimes: sheltered (East, “E”) and exposed (West, “W”). Red dots represent sampling sites within each atoll.

The Lakshadweep Archipelago is a chain of coral atolls situated in the northern Indian Ocean off the west coast of mainland India (Fig. 5a). Lakshadweep comprises 12 coral islands and submerged banks (Fig. 5b). The coral atolls of Lakshadweep have shallow lagoons and are surrounded by barrier reefs^105^. The southwest monsoon and north-south orientation of most atolls have given rise to distinct windward and leeward aspects, which strongly influence the ecology and geography of Lakshadweep islands^52,67^. Although fishing has been the mainstay of the people of Lakshadweep, commercial fisheries have primarily been centred around the targeted fishing of pelagic skipjack tuna (*Katsuwonus pelamis*), leading to relatively undisturbed fish communities in the coral reefs^57^. However, commercial reef fishery is on rise in the archipelago, with different atolls of Lakshadweep experiencing various degrees of fishing pressure^71^.

We sampled three atolls of the Lakshadweep Archipelago: Bitra, Kadmat, and Kavaratti (Fig. 5c, d and e). The islands represent a gradient of piscivorous fish density, with Bitra at the higher and Kavaratti at the lower end of the spectrum. Previous work from Lakshadweep suggests this gradient to be fishing-induced rather than natural^71^. This shift is most prominent in the atoll of Kavaratti, which had the highest fish biomass before reef fisheries became a mainstay on the island^106^. The island fishery is mainly carried out using the traditional hook and line method, which selectively harvests mesopredatory fishes from the reef, leaving the other functional groups, including herbivores, relatively undisturbed^57,71^. In addition to the fishing-induced biomass gradient, in Bitra, we were able to sample a spawning aggregation site of *Plectropomus areolatus* (squaretail grouper) during spawning events. Spawning aggregations represent very high densities of fish biomass and can have long-lasting effects on nutrient dynamics and primary productivity in the aggregating reef, even after the aggregation is over^11,26^. The aggregation in Bitra occurs around every new moon period from November to April, potentially creating a hotspot of nutrient enrichment in these reefs throughout this period. Thus, we included the site in our mesopredator biomass gradient. We sampled 11 sites across the three islands: two in Bitra, five in Kadmat, and four in Kavaratti (Fig. 5c, d and e). In Bitra, the sites were located on the eastern (sheltered) aspect of the lagoon; in Kadmat, three sites were located on the east (sheltered), and two were on the west (exposed); and in Kavaratti, two sites were located on the east (sheltered), and two were on the west (exposed). We collected all data for the current study between January 2024 and May 2024.

### Field assessments

#### Piscivore and herbivore fish community

We characterized the piscivorous and herbivorous fish community in each reef using underwater visual census (UVC). Three belt transects, each having a dimension of 50 m × 10 m (500 m^2^ area per transect), were sampled at 12–14 m depth. A minimum distance of 10 m was maintained between two consecutive transects to ensure independence. Two observers of comparable experience conducted the UVCs. The observers swam parallel to each other along the transects. One observer noted the mesopredatory fishes belonging to the families Serranidae, Haemulidae, Lethrinidae, Lutjanidae, Cirrhitidae, Malacanthidae, Aulostomidae, Holocentridae, Fistularidae and Labridae (Supplementary Material 8b). Apex predators, such as sharks, are extremely rare in the reefs of Lakshadweep, and thus data on their very rare occurrences (one transect out of 33 transects across 11 sites) were not considered for the this study. The other observer noted herbivorous fish species belonging to the families Scaridae, Kyphosidae, Acanthuridae and Siganidae (Supplementary Material 8a). Only individuals with total body length greater than 10 cm were noted during UVCs. Most observed fish individuals had a total body length between 10 cm and 65 cm (Supplementary Material 7a, 7b). We identified all the individuals on the transect to the species level and noted their abundance and size (total length) to the nearest centimetres. All the surveys were conducted between 0900 h and 1200 h.

#### Algal cover and structural complexity

Reef substrates were photographed every 10 m on each belt transect (*n* = 6 in each transect) with a standard reference object in the frame. The images were analyzed using the software ImageJ to estimate the percentage of turf algal cover within a 1 m × 1 m area^107^. We averaged the estimates from the three transects to obtain the percentage algal cover of the reef. We measured structural complexity as the average vertical height of the reef on the belt transects. We took measurements at every 5 m distance (*n* = 10 in each transect). Estimates from the three transects were averaged to obtain the structural complexity of the reef^84^. Previous studies from Lakshadweep have found that vertical canopy height was highly correlated with other conventional measures of structural complexity, such as rugosity^58^.

#### Algal growth rates through herbivore exclusion

We established herbivore exclusion cages (20 cm × 20 cm × 20 cm) to quantify algal growth. Box-shaped cages were constructed using plastic mesh (mesh size 2 cm diameter) and cable ties. We selected areas of rigid substrate at a depth of 12–14 m on the reef that were covered with algal turfs while avoiding farming damselfish territories and heavily sediment-laden substrates. The cages were operational for only 14–21 days and, therefore, did not accumulate significant amounts of fouling algae over the experimental period. We set up four cages in each site and maintained a minimum distance of 10 m between two adjacent exclosures (*n* = 44). The exclosures were attached to the reef substrate directly using cable ties. The cages ensured that herbivorous reef fishes were excluded from feeding within the enclosed area. However, the mesh size allowed smaller-sized herbivorous fishes and other grazing invertebrates (< 2 cm width) to enter the cages and ensured that light and water flow were not impeded. Herbivorous urchins were also excluded from grazing inside the exclosures; their abundance is typically low in the reefs of Lakshadweep and their contribution towards total herbivory is likely negligible^105^.

To quantify algal growth rates, the height of 10 fronds of turf algae was measured underwater using the depth probe of a vernier calliper on the day of cage installation. The depth probe yields the exact distance between the tips of the calliper. This distance between the tips was recorded by imprinting it on a saltwater-resistant pressure-sensitive poster adhesive (blue tac) attached to an acrylic board. The imprint was later measured on the surface using a digital vernier calliper, and the values up to one decimal point were noted down in millimeters^108^. The process was repeated the day the cages were removed from the reefs. The average algal height difference between the first and final days was divided by the number of days the exclosure was operational in the reef to obtain daily algal growth rate. As initial turf length is known to affect turf growth^109^, we calculated proportional turf growth (change in turf height per month/initial height of turf) for each exclosure. The value was multiplied by 30 to obtain proportional algal growth rate for a month.

#### Herbivory rates through in-situ exclosures

A 20 cm × 20 cm area was marked using a fishing buoy adjacent to the herbivore exclusion cages and served as an open herbivory plot (*n* = 44). A minimum distance of 1 m was maintained between the exclosure and the control plot to avoid cage effects. The height of 10 turf algal fronds was measured using the same method as inside the cages. Daily herbivory rate was estimated as the difference between daily algal growth inside the exclosures and the paired open herbivory plot present next to it at the end of the period. The value was multiplied by 30 to get the herbivory rate in mm/month.

#### Anti-predatory behaviour through focal observations

Across the mesopredator biomass gradient, we carried out focal observations on a candidate fish species of lower trophic level, *Ctenochaetus striatus*, to test the presence of non-consumptive effects of predators on prey fish species. While the functional role of *C. striatus* is keenly debated, with recent studies establishing the species as a detritivore rather than a herbivore^110,111^, we selected it as our candidate prey species as it is one of the most abundant primary consumers in the reefs of Lakshadweep, exhibits a similar life history, body structure and trophic position as other Acanthurid herbivores, and has been demonstrated to be prey for mesopredators in its juvenile life stages^67,84,112,113^. We expected that the anti-predatory behavioural responses of *C. striatus* individuals to predation threat are likely to be generalizable to other primary consumers in the reef – the trophic guild that the herbivorous fishes are part of^114^.

Sampling was conducted during the day between 0900 and 1200 when fishes are known to be active, and all the sampled individuals were chosen opportunistically. We followed 10 *C. striatus* individuals at each sampling site (*n* = 110). *C. striatus* individuals with body sizes (total length) greater than 18 cm were sampled to avoid behavioural variations due to body size dissimilarity. This body size is also speculated to be out of direct predation threat by coral reef mesopredators and is much greater than the average prey size of coral reef fishes^70,86^. Thus, they are deemed suitable for examining the non-consumptive effects of mesopredators on herbivore behaviour.

The focal individuals were filmed for 4 min. Consecutive observations were separated spatially by at least 10 m to avoid sampling the same individuals. We swam in one direction between two successive focal follows to avoid repeated observations of the same individual. All the observations were conducted within a narrow depth range of 10–13 m.

The videos were analyzed later to observe the proportion of time spent in vigilance by the individual. Our observations indicated that individuals resumed feeding within 30 s of video recording, and did not show any signs of diver-induced disturbance like accelerating away from the observer or repeated hiding. Hence, we excluded the first 30 s of the video. If the fish could not be seen because it had moved behind a big boulder after 30 s, we waited until it could be seen again in the video and restarted analyzing the video. The videos were analyzed for a total of 3-minutes. Time spent in vigilance is widely considered to be a reliable metric of understanding anti-predatory response of prey to predation threat, with increasing predation threat being associated with an increased amount of time spent in vigilance by prey individuals^115,116^. Thus, we noted down time spent in vigilance by the *C. striatus* individuals during the 3-minute. Following the existing literature, vigilance was defined as the behavioural state in which the focal individual swam with its face pointed towards the water column at a upward angle away the reef substrate (Fig. 4b)^117^.

### Quantification and statistical analysis

#### Quantifying mesopredatory and herbivorous fish biomass

We used fish identity, body size and abundance data obtained from UVCs to estimate the biomass using the formula W = a × L^b^, where W = estimated biomass, L = observed length of the fish, and a and b values are standard fish values obtained for each species from Fishbase^118^. Total biomass at the level of the transect was divided by the area of each transect to obtain fish biomass per unit area. A mean of the transect-level values was estimated to obtain site-level fish biomass per unit area.

Previous studies suggest the existence of a fishing-induced mesopredatory fish biomass gradient in the atolls of Lakshadweep^71^. Our underwater visual census data also suggested the presence of a mesopredator biomass gradient across the 11 sampled sites in Lakshadweep. We also found a strong positive correlation between mesopredator biomass and herbivore biomass (*r* = *0.9*). Mesopredator and herbivore biomass were thus log-transformed to account for the parametric correlation between the two variables and avoid multicollinearity issues in subsequent models (*r = 0.65*).

#### Controlling for pelagic nutrient subsidies

The oceanic coral reefs typically thrive in phosphorus-limited seascapes, and pelagic nutrients vectored by planktivorous fishes and piscivorous fish excretion are considered to be the two major sources of phosphorus in such systems^11,66,68,69,119,120^. Numerous studies have established planktivore-mediated pelagic phosphorus input as a ubiquitous and salient source of nutrients in offshore coral reef ecosystems^69,121^. In this study, we were unable to estimate pelagic phosphorus input in the reefs of Lakshadweep. However, studies on plankton biomass and hydrodynamics suggest that pelagic plankton transport to coral reefs through subsurface waters is mediated by wind and wave exposure^68^. The north-south orientation of Lakshadweep’s atolls gives rise to two distinct wave exposure regimes: the calmer and sheltered east, or the leeward aspect, and the turbulent and exposed west, or the windward aspect^52,67^. On average, the windward western aspect of Lakshadweep atolls experiences about three times greater wave power across the season compared to the leeward eastern aspect, with the contrast being highest during the Indian summer monsoon^122^. These contrasting exposure regimes likely translate to differences in pelagic nutrient subsidies. Thus, to statistically control for pelagic nutrient subsidies, we included the physical aspects of the sites in our regression models.

### Productivity along mesopredator biomass gradient

#### Estimating consumer-derived nutrient inputs

We used data obtained from UVCs to estimate consumer-derived nutrient input in the water. We used a global consumer-derived nutrient input dataset to calculate size-specific nitrogen and phosphorus input in the system for each species of herbivore and piscivore fish^123^. If the nutrient input value for any observed body size for a species was unavailable, the nearest available body size (difference of ≤ 2 cm) in the dataset was considered to calculate the nutrient input. If the values were not present for any particular species, the values available for a congeneric species were considered for the calculation^98^. We converted the absolute mass of nitrogen and phosphorus input to moles by dividing each by the atomic mass of the respective element. The N: P molar ratio (ratio between the amounts of elements in moles) was calculated for all the sampled sites. We estimated the mean N: P molar ratio and calculated the standard error using a non-parametric bootstrapping method with 2000 iterations to infer the relative role of herbivores and piscivores in supplying nutrients in the system.

#### Estimating algal growth rates

We employed linear mixed-effects models to investigate the impact of herbivore and piscivore biomass on algal growth rates. Proportional algal growth rate was modelled against log-transformed mesopredator and herbivore biomass. Aspect was added as a fixed effect in the model. Site identity was included as a random intercept to account for any other factors at the site level that influence algal growth rates. Both continuous predictors were scaled before being included in the model.

#### Quantifying herbivore productivity

Recent modelling advancements have enabled the calculation of productivity of reef fish assemblages by combining underwater census data with predicted growth and size-based mortality rates^92,124^. Thus, we calculated herbivorous fish productivity using fish species identity, size and abundance obtained from the underwater visual census^124,125^. The census data was filtered only to include grazers, scrapers and excavators - the herbivore functional groups that feed on turf algae. We estimated the standardized growth parameter, *K*_*max*_, for all individuals using observed body size, diet and a mean sea surface temperature of 28 °C ^124^. Using the estimated species and size-specific *K*_*max*_ values, we estimated the age of the individuals using the individual age framework and estimated biomass gain through somatic growth over a day for all the individuals^124,126^. Finally, we subtracted per-capita biomass loss due to natural mortality, calculated by combining observed fish size, species-specific maximum growth rate (*K*_*max*_) and species-specific asymptotic maximum body size. Thus, we estimated productivity as the biomass gained per day by all surviving individuals at the level of the transect. We converted the value per transect to productivity per unit hectare of area per day. Productivity was modelled as a function of mesopredator biomass, resource availability, structural complexity and aspect of the site using a generalized linear mixed-effects model with gamma error distribution and log-link. Resource availability was quantified as the product of site-level percentage algal cover and average daily absolute algal growth rate obtained from the exclosures. We used site identity as a random intercept to account for any inherent variability in productivity within a site. Mesopredator biomass was log-transformed and all the continuous predictors were scaled before being used in the model.

### Herbivory rate along mesopredator biomass gradient

#### Estimating reef-wide herbivory rates

Herbivory rates obtained from the in-situ exclosures were modelled as a function of log-transformed mesopredator and herbivore biomass using a linear mixed-effects model. Herbivore biomass comprised the total biomass of grazers, scrapers and excavators, as they are known to feed on turf algae, thus contributing to herbivory^91,110^. Aspect was included as a fixed effect in the model, as herbivory rates are known to be influenced by wave exposure regimes^67^. Site-level percentage cover of turf algae was added as a fixed effect, as the availability of algal resources can determine the levels of herbivory in any unit area of the reef. We used site identity as a random intercept in the model. Both continuous predictors were scaled before being used in the model.

#### Quantifying community-level herbivory

We estimated monthly community-level herbivory on turf algae in terms of grams of carbon ingested per unit area by the herbivorous fish community present at the site. Herbivory rates were estimated by combining underwater visual census data of herbivores with a global carbon ingestion dataset^123^. We calculated size- and species-specific daily carbon ingestion by herbivorous reef fishes belonging to the functional groups: grazer, scraper and excavator. If the ingestion value for any observed size of a species was missing from the dataset, the value for the nearest body size (with a difference of ≤ 2 cm) was used to calculate herbivory rates. If a species from our study area was absent from the dataset, we used data from a congeneric species that was present in the dataset. We calculated the mean herbivory rate at the level of each transect and converted it to grams of carbon ingested per unit square meter of the reef per day. We modelled community-level herbivory rates as a function of log-transformed mesopredator biomass, percentage algal cover, structural complexity and aspect of the site using a generalized linear mixed-effects model with gamma error distribution and log-link. We used site identity as a random intercept and scaled all the continuous predictor variables before using them in the model.

### Assessing anti-predatory behaviour

Time spent in vigilance was divided by the total observable time to obtain the proportion of time spent in vigilance. We used generalized linear mixed-effects models with a beta error distribution and logit link function to model proportion of time spent in vigilance. Log-transformed mesopredator and herbivore biomass were added as predictor variables. Resource availability was quantified as the product of site-level proportion of algal cover and mean daily absolute algal growth rate (mm) obtained from the exclosures and was added as a fixed effect. Aspect was added as a fixed effect to account for wave exposure regime. Structural complexity was added as a predictor. Site was added as a random intercept to account for any inter-site variability. All continuous predictors were scaled before being used in the model.

### Validity and diagnostics of models

Models were fitted based on the distribution of the data as revealed by the preliminary analyses. The model fit for linear mixed-effects models was visually examined using a plot of model residuals against the fitted values to check for homoskedasticity, and a Q-Q plot and histogram of the model residuals to check for normality. The Shapiro-Wilk test for normality was used on the residuals of the datasets that were difficult to assess visually for normality. The Variance Inflation Factor (VIF) was calculated for each fitted model, ensuring the absence of multicollinearity between predictors. Model fit for generalized linear mixed-effects models with beta and gamma error distribution was assessed using plots of raw residuals, Pearson’s residuals and deviance residuals, a plot of simulated residuals against the model residuals and a Q-Q plot of residuals. All models were checked for the presence of overdispersion.

All analyses were performed using R version 4.4.2^127^. Package *“lme4”* was used to run linear mixed-effects models^128^. Package *“glmmTMB”* was used to run generalized linear mixed-effects models with beta and gamma error distributions^129^. Package *“car”* was used to check for the variance inflation factor^130^. Package *“DHARMa”* was used to check the model fit of the generalized linear mixed-effects models with beta and gamma error distribution^131^. Package *“visreg”* was used to extract data from models^132^, and the package *“tidyverse”* was used for data cleaning and visualization^133^. Package *“performance”* was used to check for overdispersion^134^. Herbivore productivity was estimated using the package *“rfishprod”*^124^.

## Supplementary Information

Below is the link to the electronic supplementary material.

Supplementary Material 1

## Data Availability

All data supporting the findings and conclusions of this article will be made publicly available in the Zenodo data repository (https://doi.org/10.5281/zenodo.15143823) upon publication. Any additional information required to analyze the data will be made available by the corresponding author upon request.

## Declarations

### Permission statement

We confirm that the required permissions to conduct field work in the atolls of Lakshadweep Archipelago were obtained from the Department of Environment and Forests, Union Territory of Lakshadweep (F. No. 1/5/2023-E&F/1045). Should you need any further documentation or information, please feel free to contact the corresponding author.
