# Supplementary material for: Predators can facilitate herbivory in nutrient-limited marine ecosystems
Source: Sci Rep. 2025 Dec 30;16:3978. doi: 10.1038/s41598-025-34145-6 (PMC12855930; doi:10.1038/s41598-025-34145-6)

Predators can facilitate herbivory in nutrient-limited marine ecosystems

Supplementary Material 1: *Linear mixed-effects model summary for proportional algal growth rate (n = 44). Estimates correspond to scaled values of the predictors.*

Formula	*Proportional Algal Growth Rate ~ Predator biomass (log) + Herbivore biomass (log) + Aspect + (1|Site)*			
Fixed effects	***Estimate***	***Std. Error***	***t value***	***p***
(Intercept)	1.05	0.10	10.56	**<0.001**
Mesopredator biomass (log)	0.41	0.11	3.64	**0.001**
Herbivore biomass (log)	-0.16	0.10	-1.72	0.094
Aspect [west]	-0.61	0.19	-3.29	**0.002**
Random effects				
Residual			*0.11*	
Site			*0.03*	
N_{site}			*11*	
Marginal R^2 / Conditional R^2			*0.39 / 0.53*	

Supplementary Material 2: *Proportional algal growth rate (n =44) was higher in the sheltered eastern aspect of the atolls compared to the exposed western aspect. The points represent the partial predicted values from the linear mixed-effects model with all other predictors set at their mean. The error bars represent the 95% confidence intervals.*

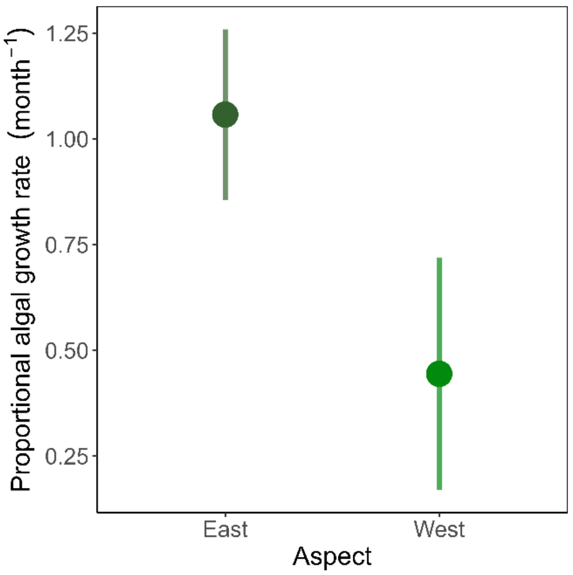

Supplementary Material 3: *Summary of the generalized linear mixed-effects model with gamma error distribution and log-link for herbivore productivity (n = 33). Estimates are untransformed and correspond to scaled values of the predictors.*

Formula	*Herbivore productivity ~ Predator biomass (log) + Resource availability + Structural complexity + Aspect + (1|Site)*			
Fixed effects	***Estimate***	***Std. Error***	***z value***	***p***
(Intercept)	5.12	0.16	32.71	<0.001
Mesopredator biomass (log)	0.34	0.14	2.47	**0.013**
Resource availability	0.22	0.13	1.66	0.097
Structural complexity	0.34	0.13	2.58	**0.010**
Aspect [west]	0.57	0.33	1.72	0.086
Random effects				
Residual	*0.26*			
Site	*0.01*			
N_{site}	*11*			
Marginal R^2 / Conditional R^2	*0.44 / 0.46*			

Supplementary Material 4: *Linear model output for reef-wide herbivory rates (n = 44). Estimates correspond to scaled values of the predictors.*

Formula	*Herbivory Rate ~ Predator Biomass (log) + Turf-feeding Herbivore Biomass (log) + Percentage algal cover + Aspect + (1|Site)*			
Fixed effects	***Estimate***	***Std. Error***	***t value***	***p***
(Intercept)	*2.79*	*0.21*	*13.794*	***<0.001***
Mesopredator biomass (log)	*0.64*	*0.26*	*2.504*	***0.017***
Herbivore biomass (log)	*-0.31*	*0.20*	*-1.507*	*0.140*
Percentage algal cover	*-0.01*	*0.19*	*-0.064*	*0.949*
Aspect [west]	*-0.72*	*0.39*	*-1.858*	*0.071*
Random effects				
Residual	*0.94*			
Site	*0.00*			
N_{site}	*11*			
Marginal R^2 / Conditional R^2	*0.20 / 0.20*			

Supplementary Material 5: *Summary of the generalized linear mixed-effects model with gamma error distribution and log-link for community-level herbivory rates (n = 33). Estimates are untransformed and correspond to scaled values of the predictors.*

Formula	*Herbivory ~ Predator Biomass (log) + Percentage algal cover + Structural complexity + Aspect + (1|Site)*			
Fixed effects	***Estimate***	***Std. Error***	***z value***	***p***
(Intercept)	*1.00*	*0.13*	*7.52*	***<0.001***
Mesopredator biomass (log)	*0.41*	*0.13*	*3.31*	***0.001***
Percentage algal cover	*-0.02*	*0.11*	*-0.17*	*0.869*
Structural complexity	*0.26*	*0.12*	*2.20*	***0.028***
Aspect [west]	*0.12*	*0.28*	*0.45*	*0.653*
Random effects				
Residual		*0.26*		
Site		*0.00*		
N_{site}		*11*		
Marginal R^2 / Conditional R^2		*0.34 / 0.34*		

Supplementary Material 6: *Summary of the generalized linear mixed-effects model with beta error distribution and logit-link for proportion of time spent in vigilance (n = 110). Estimates are untransformed and correspond to scaled values of the predictors.*

Formula	*Vigilance Time ~ Predator biomass (log) + herbivore biomass (log) + Resource availability + Structural complexity + Aspect + (1|Site)*			
Fixed effects	***Estimate***	***Std. Error***	***z value***	***p***
(Intercept)	-1.25	0.43	-2.92	0.004
Mesopredator biomass (log)	0.06	0.13	0.47	0.637
Herbivore biomass (log)	0.01	0.10	0.07	0.945
Resource availability	0.01	0.11	0.05	0.964
Structural complexity	-0.01	0.01	-1.23	0.218
Aspect [west]	0.10	0.17	0.57	0.566
Random effects				
Residual		*-0.07*		
Site		*0.00*		
N_{site}		*11*		
Marginal R^2 / Conditional R^2		*0.18 / 0.18*		

Supplementary Material 7: *Frequency distribution of observed herbivorous (a.) and mesopredatory (b.) reef fish body sizes across all the sampled sites. The black dashed lines represent the median body sizes (20cm for herbivores, 30cm for mesopredators) for the two groups in our study.*

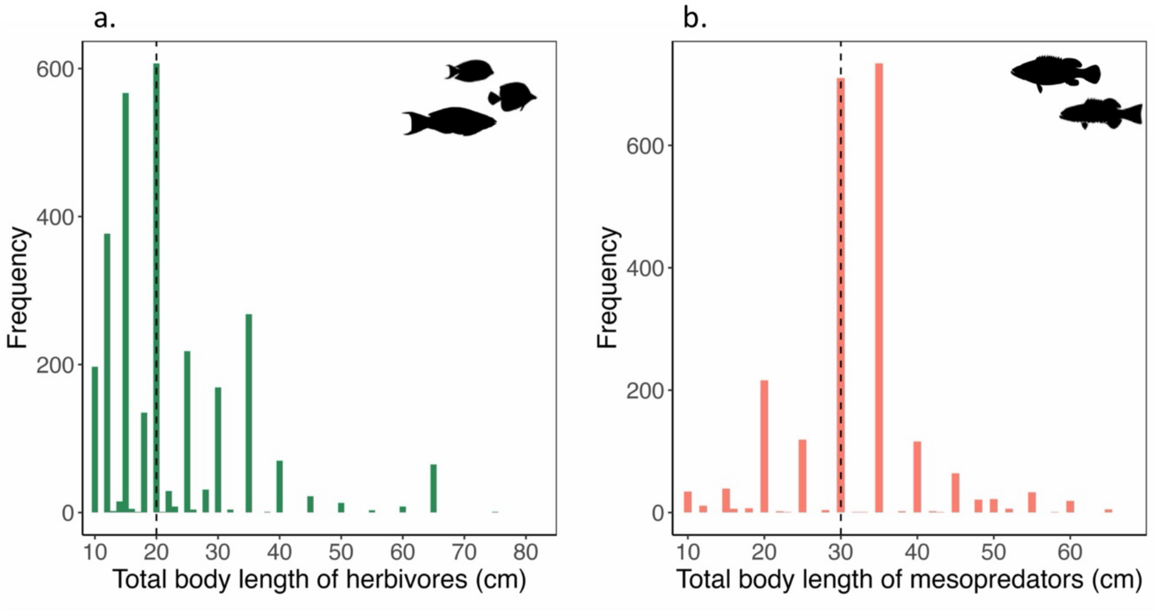

Supplementary Material 8: *Frequency distribution of observed herbivorous (a.) and mesopredatory (b.) reef fish families across all the sampled sites.*

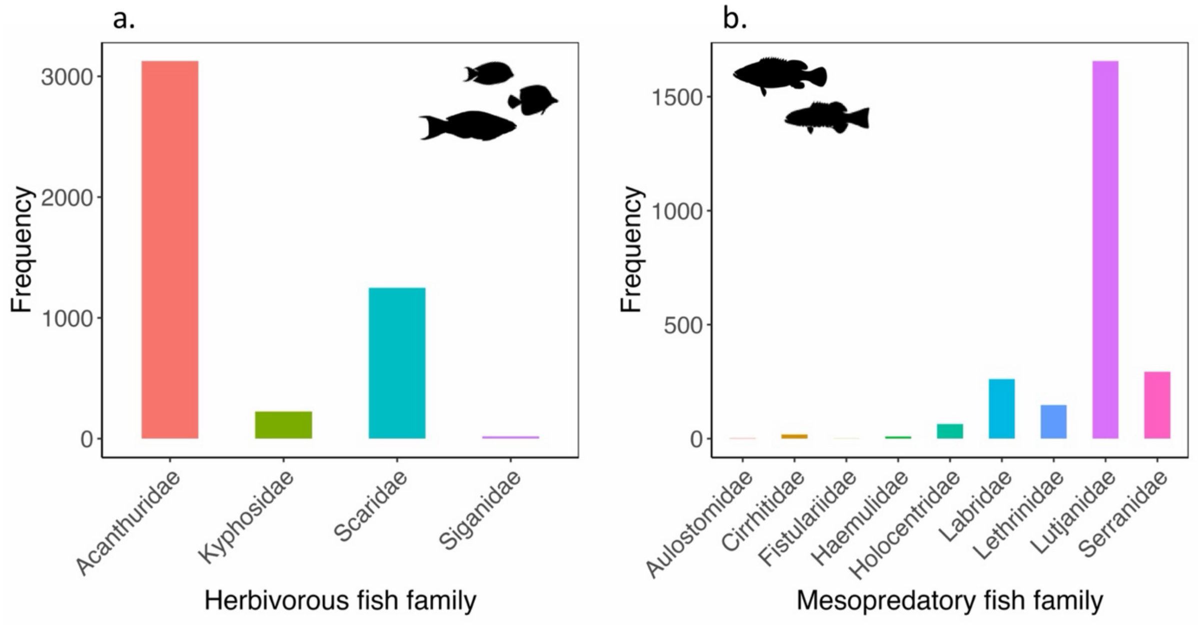

Supplement: Supplementary file 1 — Supplementary Material 1 [file 41598_2025_34145_MOESM1_ESM.pdf]
